# Protecting your skin: a highly accurate LSTM network integrating conjoint features for predicting chemical-induced skin irritation

**DOI:** 10.1186/s13321-025-00980-y

**Published:** 2025-03-27

**Authors:** Huynh Anh Duy, Tarapong Srisongkram

**Affiliations:** 1 Graduate School in the Program of Research and Development in Pharmaceuticals, Faculty of Pharmaceutical Sciences, Khon Kaen University, Khon Kaen, 40002 Thailand; 2 Division of Pharmaceutical Chemistry, Faculty of Pharmaceutical Sciences, Khon Kaen University, Khon Kaen, 40002 Thailand

**Keywords:** Deep learning, Skin irritation, Recurrent neural network, Gated recurrent unit, Long short-time memory, Toxicity prediction, Conjoint fingerprints, Cheminformatics

## Abstract

Skin irritation is a significant adverse effect associated with chemicals and drug substances. Quantitative structure-activity relationship (QSAR) is an alternative method bypassing *in vivo* assay for filling data gaps in chemical risk assessment. In this study, we developed QSAR models based on recurrent neural networks (RNNs) to classify skin irritation caused by chemical compounds. We utilized chemical language notation, molecular substructures, molecular descriptors, and a combination of these features named conjoint fingerprints for model construction. A simple RNN, long short-term memory (LSTM), bidirectional long short-term memory (BiLSTM), gated recurrent units (GRU), and bidirectional gated recurrent units (BiGRU) architectures were used to build the QSAR models. We found that the LSTM and a combination of molecular fingerprints and descriptors outperformed the other models significantly with 80% accuracy, 60% MCC, and 85% AUC for the external test set evaluation. Thereby, we selected this model for generalizability testing with other test sets beyond our study, ensuring that the model can be used with other data sets. Furthermore, the applicability domain of the purposed model was developed, enabling a trustable prediction will be made for a test compound. This model was developed based on OECD guidelines for skin irritation assessment and QSAR model development, assuring compliance with all required standards. The models and source codes developed in this study are publicly available, facilitating chemical design and safety evaluation, particularly for assessing the skin irritation potential of chemicals.

**Supplementary Information:**

The online version contains supplementary material available at 10.1186/s13321-025-00980-y.

## Scientific contribution

This study advances the field by developing RNNs-based QSAR models through the innovative integration of chemical language notations with conjoint molecular features for skin irritation prediction. This approach surpasses existing methods in terms of predictive performance and generalization, achieving a more robust and accurate predictive model. The public availability of models and source codes promotes reproducibility and facilitates safer chemical evaluation, distinguishing this work from prior studies.

**Supplementary Information:**

The online version contains supplementary material available at 10.1186/s13321-025-00980-y.

## Introduction

Skin irritation poses significant challenges in dermatology and pharmaceutical innovation, impacting both patient safety and treatment effectiveness. Skin irritation test data are integral to regulatory compliance in the United States (U.S.), as chemical regulatory bodies require these assessments to inform product hazard labeling and to evaluate potential risks associated with exposure to skin-irritating substances [1]. Traditionally, acute dermal irritation testing has relied on animal models in accordance with the guidelines from the Organisation for Economic Co-operation and Development (OECD TG 404) [2] and the Environmental Protection Agency (EPA OPPTS 870.2500) [3]. However, ethical concerns surrounding animal welfare have spurred significant advancements and advocacy in the development of alternative, non-animal methods for toxicity evaluation.

The Interagency Coordinating Committee on the Validation of Alternative Methods (ICCVAM), a coalition of U.S. Federal regulatory and research entities, is dedicated to validating and advancing alternative testing approaches with the principal goal of “reduce, refine, or replace the use of animals in testing where feasible” [4]. To enhance scientific reliability and improve the relevance of these alternative methods to human health, ICCVAM has developed a strategic roadmap that emphasizes the adoption of innovative methodologies for assessing the safety of chemicals and medical products [1]. *In silico* computational approaches are increasingly recognized as viable alternatives to conventional experimental procedures, addressing key knowledge gaps, strengthening regulatory decision-making frameworks, and facilitating more ethical and efficient evaluation processes. Furthermore, the U.S. Food and Drug Administration’s (FDA) current regulatory policies encourage the integration of new alternative methods, including *in silico* models, thereby fostering confidence in their applicability for safety evaluations and regulatory acceptance [5].

Within *in silico* predictive methodologies, quantitative structure-activity relationship (QSAR) modeling stands as one of the most widely applied mathematical modeling approaches for assessing the potential bioactivities of chemicals using available data from the literature [6–9]. This method also benefits toxicity assessments in various toxicity endpoints as demonstrated in the previous quantitative structure-toxicity relationship (QSTR) studies [10–12]. With the high availability of existing experimental data, the OECD organization has created a detailed guidance document on the validation of QSAR models [13]. This guideline outlines fundamental principles and provides a systematic framework for the exhaustive validation of QSAR methods for various applications, thus enhancing the trustworthiness and acceptance of these models in both regulatory and research contexts.

In recent years, machine learning (ML) techniques have emerged as promising alternatives to traditional QSAR methods, offering improved predictive capabilities through the analysis of complex biological data and chemical fingerprints [14, 15]. Additionally, advancements in deep learning (DL), particularly in recurrent neural networks (RNNs), have shown promise in enhancing the accuracy of QSAR models [16, 17]. Neural network architectures such as long short-term memory (LSTM) networks [18], bidirectional long short-term memory (BiLSTM) networks [19], gated recurrent unit (GRU) [20], and bidirectional gated recurrent unit (BiGRU) [20] have demonstrated remarkable performance in computer-aided disease diagnosis and treatment [21]. LSTM networks effectively learn long-range dependencies in sequential data by utilizing specialized memory cells to mitigate the vanishing gradient problem of simple RNN. BiLSTM networks enhance this memory cell further by processing data in both forward and backward directions, capturing context from both past and future sequences [22]. GRU networks streamline the LSTM architectures by combining the forget and input gates into a single update gate, resulting in faster training times while maintaining competitive performance. Similarly, BiGRU architectures leverage bidirectional processing to enhance learning from both the forward and backword directions of the input sequences. These five architectures process and store information through distinct pathways [23], making them vulnerable to be test for QSAR modeling.

**Fig. 1:**
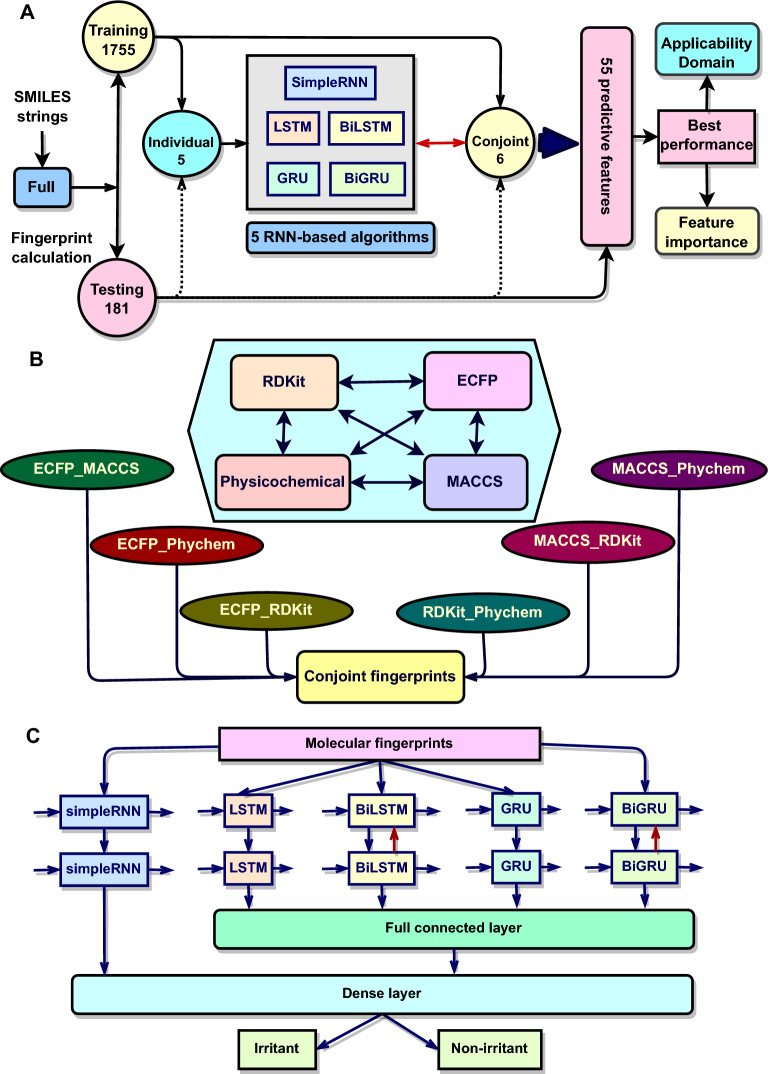
Schematic diagram of recurrent neural network (RNN)-based QSAR model development.** A** QSAR model pipeline.** B** Conjoint fingerprints construction.** C** RNN architectures for QSAR modeling

In this paper, we aim to explore the application of simple RNN, LSTM, BiLSTM, GRU, and BiGRU models in predicting skin irritation. By integrating advanced DL techniques with QSAR principles and employing both individual and conjoint features, we aspire to improve the accuracy and efficiency of skin irritation assessments, ultimately contributing to safer pharmaceutical formulations and better patient outcomes. Notably, incorporating conjoint features into QSAR models is vital for enhancing predictive accuracy. Individual features represent specific molecular characteristics, such as atomic environment or functional groups, while conjoint features capture chemical representations from both individual features and demonstrate the interactions between these individual attributes. By utilizing these features, QSAR models may better understand the complexities of skin irritation responses, thereby leading to more reliable prediction outcomes. The schematic workflow of the models construction is illustrated in Fig. 1A. Noteworthy, the major contributions of this paper are as follows:

1. We engineered 55 innovative predictive models employing RNN-based algorithms specifically tailored for the skin irritation endpoint. These models leverage six individual molecular features alongside five conjoint molecular features, capturing a comprehensive array of physicochemical descriptors, atomic environments, predefined substructures, topological properties, and character-level tokenization of SMILES strings.
2. We established robust classification models dedicated to accurately assessing skin irritation toxicity, ensuring reliability through rigorous evaluation metrics.
3. We defined a well-founded applicability domain (AD) for our most promising model by calculating the Euclidean distance between new predictors and a *k*-subset of the training data, thereby enhancing the model’s trustability.
4. We evaluated feature importance influencing model performance using the permutation feature importance technique, yielding critical insights into the molecular characteristics that drive toxicity predictions and informing the design of safer chemical compounds.
5. We tested the top promising model for its performance and generalization across various skin irritation test sets. We demonstrate that our method outperformed existing models, securing very high performance and generalizability.

## Methods

### Data set preparation

The data set employed in this study was sourced from a prior investigation and has undergone a rigorous selection and curation process from well-established databases [24]. This rigorous approach ensures a globally representative data set, wherein chemical compounds were systematically classified according to the Globally Harmonized System (GHS) into three categories: Class 1 (corrosive), Class 2 (irritant), and NC (non-classified). In our study, only Class 2 and NC compounds were kept and assigned a binary label of 1 and 0, respectively. As a result, 2,488 records were obtained including the Australian Hazardous Chemical Information System (AU HCIS) (140 records) [25], the European Registered Substances Factsheets (EU REACH) (1149 records) [26], the Japanese Chemical Risk Information Platform (JP CHRIP) (369 records) [27], the Korean National Chemicals Information System (KR NCIS) (25 records) [28], the New Zealand Chemical Classification and Information Database (NZ CCID) (580 records) [29], the Hazardous Substances Data Bank (US HSDB) (45 records) [30], the EU CLP Harmonized Classification (144 records) [31], and ChemSkin (36 records) [32]. Moreover, we have observed that the three major databases (namely AU HCIS, EU REACH, and JP CHRIP) have undergone meticulous preprocessing to ensure data integrity and analytical reliability. Of the 2,488 extracted records, 1,338 were tested on rabbits following OECD Guideline Test No. 404, while 1,150 records were based on *in vivo* data obtained using other guidelines.

All compounds were appended with SMILES strings, which are utilized for subsequent data preprocessing. Subsequently, we transformed the SMILES strings into Canonical SMILES. Then, a total of 552 duplicate compounds were systematically removed from the data set. Additionally, no inorganic compounds or mixtures were identified [33]. Following the data preprocessing phase, a total of 1,936 compounds were acquired and finally divided into training and test sets at a 9:1 ratio.

### Molecular feature encoding

#### Individual molecular descriptors

To capture a comprehensive range of chemical features, we employed an extensive sets of molecular descriptors comprising five distinct types of fingerprints: extended-connectivity fingerprints (ECFP) using a radius of 10 and a bit length of 4,096, MACCS keys using 167 bits, RDKit fingerprints using 2,048 bits, SMILES-based token representations, and a broad array of physicochemical descriptors. The physicochemical descriptors covered molecular weight (MolWt), partition coefficient (LogP), hydrogen bond donors (NumHDonors) and acceptors (NumHAcceptors), topological polar surface area (TPSA), number of rotatable bonds (NumRotatableBonds), count of aromatic rings (NumAromaticRings), count of saturated rings (NumSaturatedRings), heteroatom count (NumHeteroatoms), total ring count (RingCount), heavy atom count (HeavyAtomCount), and aliphatic ring count (NumAliphaticRings). We computed all of these features by using RDKit Python package (v.2024.9.4) [34].

#### Conjoint molecular descriptors

The conjoint fingerprints were constructed by selecting the most effective individual fingerprint to enhance predictive performance of the QSAR models (see Result). Specifically, we combined the ECFP, MACCS keys, RDKit fingerprints, and physicochemical descriptors to yield six distinct conjoint fingerprint sets: MACCS keys with physicochemical descriptors (MACCS_Phychem), MACCS keys with RDKit (MACCS_RDKit), RDKit with physicochemical descriptors (RDKit_Phychem), ECFP with physicochemical descriptors (ECFP_Phychem), ECFP with MACCS keys (ECFP_MACCS), and ECFP with RDKit (ECFP_RDKit) (Fig. 1B). Notably, SMILES tokens were excluded from the conjoint feature generation due to their incomprehensive predictive correlation with experiment values. The selection of conjoint features enabled the construction of a refined and highly effective feature sets for our predictive models.

### Simple RNN models

The simple RNN models were constructed with a single RNN layer comprising input, hidden, and output components (Fig. 1C). The hidden layer consisted of 64 units, while the output layer contained a single neuron utilizing a sigmoid activation function with a classification threshold of 0.5. Model training was conducted with a learning rate of 0.001. A binary cross-entropy was used as a loss function for error feedback optimization. The data set was partitioned into training and validation subsets with a 70:30 ratio to ensure a robust model optimization. The Adaptive Moment Estimation (Adam) optimizer was implemented to facilitate efficient gradient descent. The models were trained over 50 epochs to achieve optimal performance.

### GRU and LSTM models

The GRU and LSTM networks were designed with two sequential stacking layers of GRU or LSTM networks, where the first recurrent layer comprised 64 cell units, followed by a second layer of 32 cell units. After the recurrent layers, a fully connected dense layer with 100 neurons was implemented with a ReLU activation function. The output layer, containing a single neuron, applied a sigmoid activation function, was set with a threshold of 0.5 for binary classification output. We used a learning rate of 0.001 and binary cross-entropy as the loss function. The data set was also divided into training and validation subsets with a 7:3 ratio to support a robust model optimization. Gradient descent was optimized using the Adam optimizer, and the models were trained over 50 epochs to achieve reliable prediction outcomes.

### BiGRU and BiLSTM models

The BiGRU and BiLSTM architectures were similarly developed with two sequential BiGRU or BiLSTM layers, where the initial layer contained 64 cell units and the subsequent layer contained 32 cell units like the GRU and LSTM models. Following these layers, a fully connected layer with 100 neurons applied the ReLU activation function and a output layer consisted of a single neuron utilizing a sigmoid activation function with a 0.5 threshold were used for model construction. The training was optimized using ADAM function with a learning rate of 0.001 and binary cross entropy. The training data was partitioned into training and validation subsets with a 7:3 split ratio. These models were trained over 50 epochs similar to the previous RNN models. We used TensorFlow [35] (v2.17.0), Scikit-Learn [36] (v1.5.2), NumPy [37] (v.1.26.4) for model development.

### ML models

This study utilized two conventional ML models, random forest (RF) and light gradient boosting machine (LightGBM), as benchmarks to evaluate predictive performance against the optimal model identified in this study. The RF classifier was employed for predictive modeling using the scikit-learn library (v1.5.2). RF algorithm is an ensemble learning method that constructs multiple decision trees during training. In this study, the classifier was initialized with 100 decision trees (n_estimators=100), which provides a balance between computational efficiency and predictive performance. To ensure reproducibility, a fixed random seed (random_state=42) was applied, controlling the randomness involved in the tree-building process.

LightGBM is a highly efficient, gradient-boosting framework designed for fast training and low memory consumption, particularly effective for handling large data sets with high-dimensional features. In this study, the classifier was configured with 31 leaves and 100 estimators, optimizing the balance between model complexity and generalization performance. Additionally, a fixed random seed (random_state=42) was applied to ensure reproducibility.

### Model evaluation

In this study, the classification outcomes are delineated into four categories: true positives (TP), true negatives (TN), false positives (FP), and false negatives (FN). These metrics serve as foundational elements for a comprehensive evaluation of the model’s performance, employing five critical assessment metrics: accuracy, specificity, sensitivity, Matthews correlation coefficient (MCC), and area under the curve (AUC). Each of these metrics is computed based on the formulations presented in eqs  1- 4, except the AUC value that was calculated based on the area under the curve between sensitivity and 1-specificity values.

1 $$\begin{aligned} & Accuracy = \frac{TP + TN}{TP + TN +FP+FN} \end{aligned}$$

2 $$\begin{aligned} & MCC = \frac{(TP \cdot TN - FP \cdot FN)}{\sqrt{(TP + FP) \cdot (TP + FN) \cdot (TN + FP) \cdot (TN + FN)}} \end{aligned}$$

3 $$\begin{aligned} & Sensitivity = \frac{TP}{TP+FN} \end{aligned}$$

4 $$\begin{aligned} & Specificity = \frac{TN}{TN+FP} \end{aligned}$$

In this study, the established acceptance criterion was set with a threshold of MCC value greater than 0.5, with an ideal target nearing 1, signifying a strong correlation between predicted and actual experimental outcomes [38]. Nevertheless, the accuracy, sensitivity, specificity, and AUC were also used to evaluate the overall accuracy, sensitivity, specificity, and the ability to discriminate the positive and negative values, respectively.

### Applicability domain

We implemented an Euclidean distance-based *k*-nearest neighbors (*k*NN) algorithm to accurately delineate the applicability domain (AD). This approach delineated the proximity between a target compound and its *k* nearest compounds in the training set [39], facilitating reliable classification of both in-domain and out-of-domain compounds. The AD criteria are articulated through eqs  5 and  6, where the $D_{i}$ quantifies the average Euclidean distance between a new prediction and its *k* nearest training data, serving as an indicator of similarity. An average Euclidean distances was calculated between each training sample and its *k* closest neighbors, yielding a clear demarcation that enhances the reliability of predictions and defines the operational boundaries of the model [40]. Within the training set, $D_{k}$ and $\sigma$ denote the mean and standard deviation of these distances among the training data, respectively. The *Z*-score governs the significance level of the AD model, with a threshold of 0.5.

For Within-Domain:

5 $$\begin{aligned} D_{i} < D_{k} + \sigma \times Z \end{aligned}$$

For Out-of-Domain:

6 $$\begin{aligned} D_{i} \ge D_{k} + \sigma \times Z \end{aligned}$$

### Permutation feature importance

Permutation feature importance is a powerful technique that significantly enhances the interpretability of predictive models by elucidating the individual contributions of features to overall model performance. This method operates by systematically disrupting the relationship between features and their corresponding outcomes through the random permutation of feature values. By evaluating the subsequent decline in model performance, we can compute the importance score that reflects the predictive efficacy of each feature as defined in eq  7. This score is derived using accuracy of original model minus the accuracy of permutation model, providing critical insights into the model’s decision-making process [39].

7 $$\begin{aligned} \text {Importance score} = \text {Accuracy (original)} - \text {Accuracy (permuted)} \end{aligned}$$

### Statistical analysis

An in-depth examination was performed to evaluate the differences in metrics across different models, facilitating the identification of the most productive model for the toxicity endpoint. This thorough statistical method guaranteed a strong assessment of model efficiency across the toxicity endpoints. The first step consisted of assessing the normality of the data with the Shapiro-Wilk test, and subsequently, evaluating the homogeneity of variance using Levene’s test. Following this, the Kruskal-Wallis test, which is a non-parametric statistical method, was utilized to assess if there were statistically significant differences in the physicochemical properties or performance metrics of the QSAR models. To delve deeper into particular group differences, Dunn’s test was utilized as a post-hoc analysis for comprehensive pairwise comparisons. The *p*-value lower than 0.05 was considered statistically significant.

## Results

### Chemical space of irritants and non-irritants

**Fig. 2:**
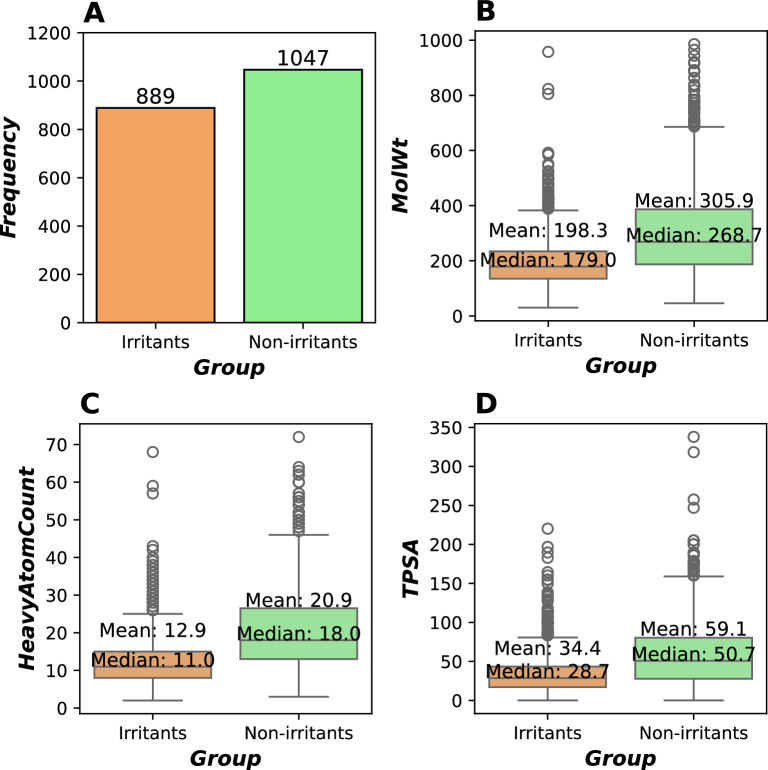
Chemical distribution between irritants and non-irritants used in this study.** A** Class distribution between irritants and non-irritants.** B** Molecular weight (MolWt) distribution between irritants and non-irritants.** C** Non-hydrogen atoms (HeavyAtomCount) distribution between irritants and non-irritants.** D** Topological Polar Surface Area (TPSA) distribution between irritants and non-irritants

We started by evaluate the physicochemical properties of the irritants (n=889) and non-irritant (n=1047) compounds as depicted in the Fig2A. The results show that irritants consistently have significantly lower molecular weights (median: 179.0 vs 268.7), number of heavy atoms (median: 11.0 vs 18.0), and topological polar surface areas (median: 28.7 vs 50.7) compared to non-irritants (*p* < 0.05), as showed in Fig2B-D. This pattern highlighted the important distinctions in physicochemical characteristics that could be vital for classifying the irritation potentials of these compounds.

**Fig. 3:**
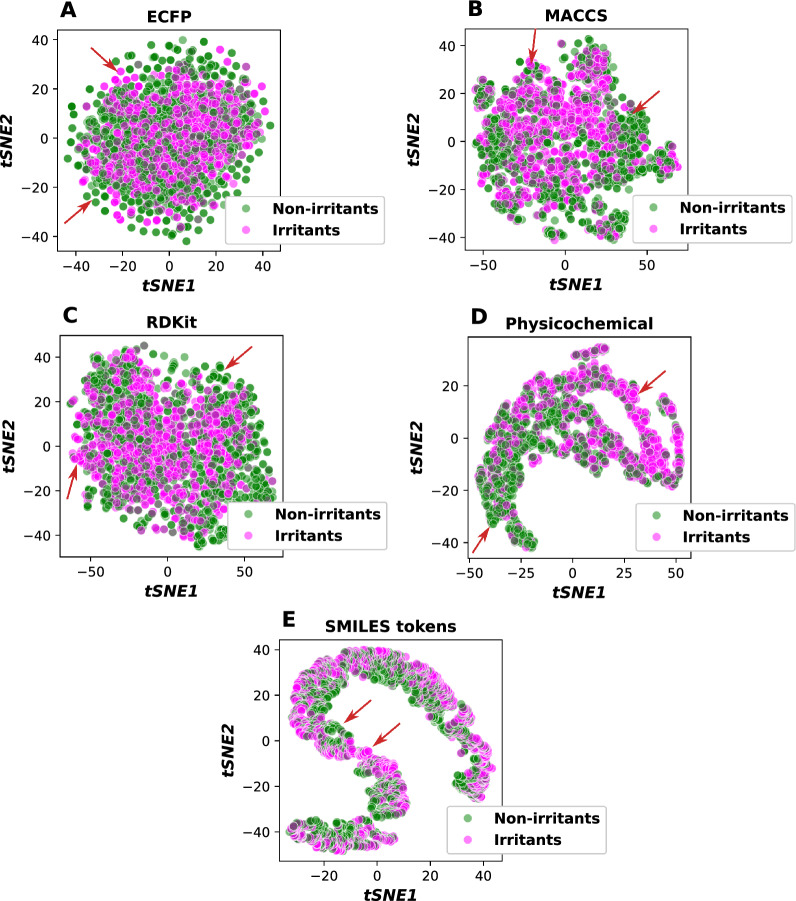
Molecular features distribution of the data set using** A** Extended circular fingerprints (ECFP),** B** MACCS keys fingerprints,** C **RDKit fingerprints,** D **Physicochemical descriptors, and** E** SMILES tokens. Red arrows indicate the unique non-overlap island of chemicals in each group

We further utilized the unsupervised t-distributed stochastic neighbor embedding (t-SNE) algorithm to visualize the chemical distribution with different molecular fingerprints and descriptors as shown in Fig3. Our analysis revealed that ECFP shows a small degree of discrimination between the irritants and non-irritants (Fig3A). However, MACCS, RDKit and physicochemical properties demonstrate a higher degree of discrimination between the irritants and non-irritants as observed in Fig. 3B-D, showcasing their potential predictive capabilities. In contrast, the representation of compounds using SMILES tokens exhibited a less distinct separation between these classifications (Fig3E). This observation prompted a deeper investigation into the influence of molecular fingerprints on the effectiveness of our predictive models, underscoring their critical role in skin toxicity assessment.

Non-linear techniques such as t-SNE and uniform manifold approximation and projection (UMAP), along with the linear method principal component analysis (PCA), are widely employed for the visualization of chemical spaces [41–43]. In this research, the t-SNE algorithm was selected for its superior ability to capture local structure and uncover intricate patterns within high-dimensional chemical space. This is crucial for accurately visualizing molecular relationships. While PCA effectively reduces dimensionality by preserving global variance, it may fail to distinctly separate complex and non-linear molecular interactions. From a different perspective, PCA optimizes the global variance in the data set, which often leads to poor preservation of local structures-such as clusters of structurally or functionally similar molecules-making it less effective for tasks like molecular similarity analysis [44, 45].

Alternatively, UMAP offers the optimal balance between preserving both the local and global structure of the data set, a capability that t-SNE lacks [41]. This method is increasingly recognized as an effective dimensionality reduction technique, capable of accurately preserving the data structure in the projected components [46]. However, it may face challenges in capturing subtle local relationships essential in chemical space, particularly for structurally similar compounds with minor variations. In some cases, UMAP may not fully capture the density variations in the data, leading to potential misrepresentations of local relationships [47]. In addition, some studies have shown that among the evaluated dimensionality reduction algorithms, all non-linear methods were effective in preserving neighborhood structures, outperforming PCA. Notably, t-SNE demonstrated superior performance in maintaining the closest neighbors [48]. Therefore, t-SNE is preferable when capturing highly localized molecular relationships and distinct chemical clusters is the priority, thereby facilitating the visual identification of meaningful molecular groupings.

### RNN models performance on the test set

**Fig. 4:**
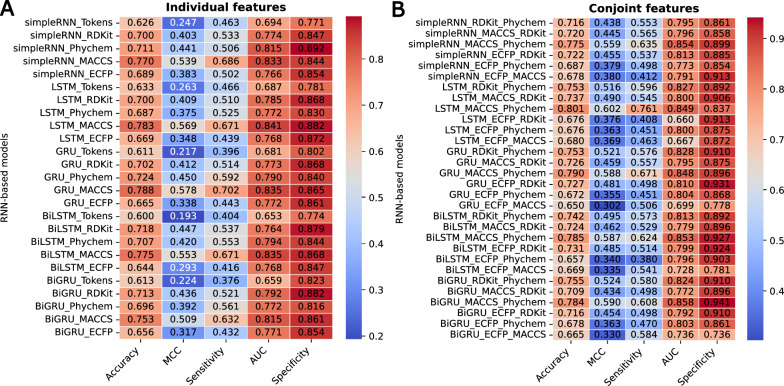
Predictive performance of RNN models for skin irritation:** A** individual features and** B** conjoint features. All metrics were retrieved from the mean of three separate experiments

We further evaluate the classification performance of the RNN-based models with individual fingerprints as illustrated in Fig.4A. The individual fingerprints demonstrated varying levels of performance metrics, including accuracy, MCC, sensitivity, AUC, and specificity, across the 25 predictive models. The metrics ranged from 0.600 to 0.788 for accuracy, 0.193 to 0.578 for MCC, 0.376 to 0.702 for sensitivity, 0.653 to 0.841 for AUC, and 0.771 to 0.892 for specificity. Notably, the GRU model utilizing MACCS key fingerprints exhibited the superior performance, attaining an accuracy of 0.788, an MCC of 0.578, and a sensitivity of 0.702 compared to the other models. These findings indicate the model’s robust capacity to accurately predict both irritants and non-irritants, resulting in an overall accuracy of 78.8% and an impressive irritant identification rate of 70.2%. The highest MCC value of this model reflected favorable outcomes across all four categories of the confusion matrix, indicating a strong correlation between the predicted and actual classifications [49]. Furthermore, a specificity value of 0.865 highlighted the model’s efficacy in detecting non-irritation compounds with a predictive rate of 86.5%. The model’s ability to differentiate between positive and negative instances was further substantiated by an AUC value of 0.835, underscoring its predictive reliability.

Among the individual molecular fingerprints, we identified and selected additional molecular fingerprints suitable for generating combined features based on their performance. Notably, models utilizing SMILES token-based representations yielded the lowest evaluation metrics with particular deficits in MCC values compared to other models. This observation aligned with the findings in chemical space (Fig3E), where SMILES tokens failed to provide effective differentiation between irritant and non-irritant chemicals. Consequently, only ECFP, MACCS keys, RDKit fingerprints, and physicochemical descriptors were chosen to construct the conjoint fingerprints.

The performance of the predictive models were significantly enhanced by developing 30 distinct models based on conjoint fingerprints (Fig4B). This approach yielded remarkable improvements in predictive performance compared to individual fingerprints, with accuracy, MCC, sensitivity, AUC, and specificity scores ranging from 0.650 to 0.801, 0.302 to 0.602, 0.380 to 0.761, 0.660 to 0.858, and 0.736 to 0.941, respectively. Integrating conjoint features enhances evaluation metrics of RNN-based models, achieving improvements of +3.5%, +13.1%, +2.6%, +3.0%, and +4.0% in average values of accuracy, MCC, sensitivity, AUC, and specificity, respectively. Importantly, the LSTM model utilizing MACCS keys in combination with physicochemical descriptors (referred to as MACCS_Phychem) demonstrated superior performance, achieving maximum values of 0.801 for accuracy, 0.602 for MCC, and 0.761 for sensitivity. Additionally, the AUC and specificity scores were commendable, reaching 0.849 and 0.837, respectively compared to the other models. We also found that the performance of LSTM with MACCS_Phychem was significantly higher than the LSTM with individual physicochemical descriptors (*p* < 0.05), indicating the higher performance of the conjoint features compared to the individual molecular features. As a result, the LSTM model employing MACCS_Phychem will be further utilized in subsequent experiments to validate its predictive performance.

**Fig. 5:**
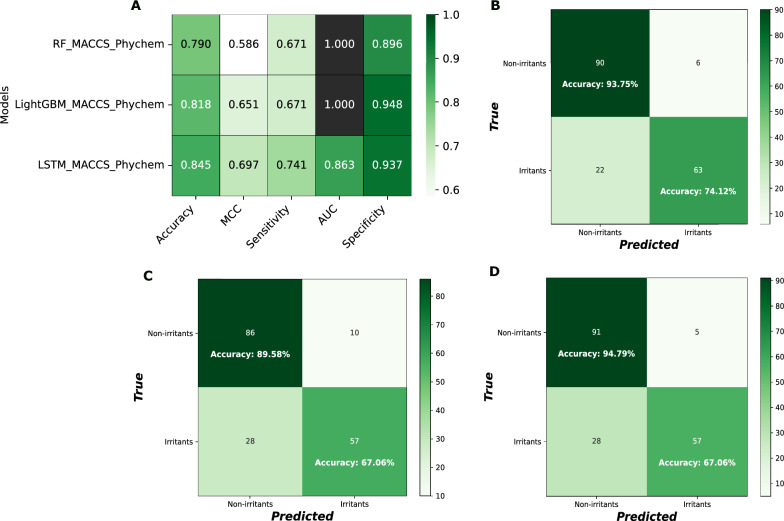
**A** Comparative performance analysis of the LSTM model against RF and LightGBM on the same data set.** B** Confusion matrix of the LSTM model with MACCS_Phychem conjoint features.** C **Confusion matrix of the RF model with MACCS_Phychem conjoint features.** D **Confusion matrix of the LightGBM model with MACCS_Phychem conjoint features. These experiments were performed with test set (n=181)

Furthermore, Fig. 5 presents a comparative analysis of the performance of LSTM, RF, and LightGBM models on the same test data set. Additionally, it displays the corresponding confusion matrices for these models, utilizing both MACCS and physicochemical descriptor-based feature representations.

Fig. 5A demonstrates that the LSTM model exhibits higher overall accuracy, MCC, and sensitivity compared to the RF and LightGBM models. The AUC and specificity values demonstrate robust model performance, though they are slightly inferior to those obtained by the other two models. The LSTM, RF, and LightGBM models correctly predicted 74.12%, 67.06%, and 67.06% of irritants and achieved a prediction rate of 93.75%, 89.58%, and 94.79% for non-irritants, respectively. The LSTM model achieves accuracy per-class ranging from 74% to 93%, confirming its robust overall performance in effectively distinguishing between irritant and non-irritant chemicals. We also can accurately trust the model on irritants prediction with 74.12% per-class accuracy, which is notably superior to the two baseline models. The corresponding results are illustrated in Fig5B–D.

**Fig. 6:**
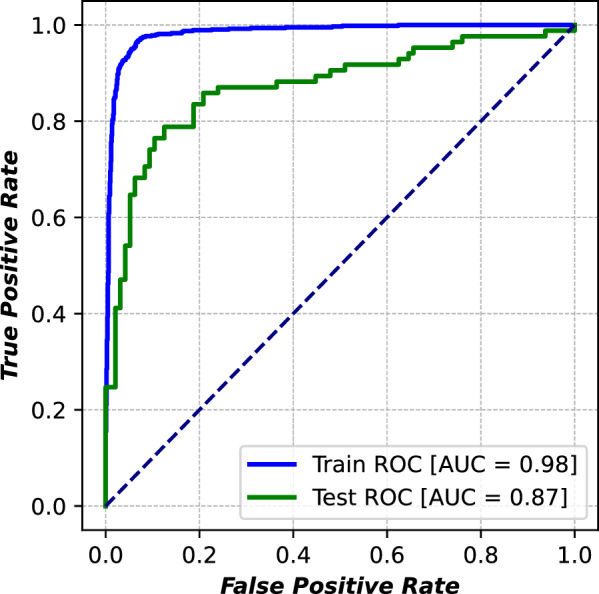
ROC-AUC plots for training and test sets of the best model

Additionally, the area under the receiver operating characteristic (ROC_AUC) graph of the best-performing model, LSTM with the MACCS_Phychem conjoint feature, as illustrated in Fig6. The model exhibited robust and reliable performance in ranking between positive and negative classes across both the training and test data sets, achieving an AUC of 0.98 on the training set and 0.87 on the test set, respectively. The consistency of performance metrics across training and test data sets indicates that the risk of overfitting was effectively minimized. This may be due to the careful tuning of batch size and learning rate to achieve a balance between training stability and generalization, as mentioned in the Methods section.

### AD analysis

**Fig. 7:**
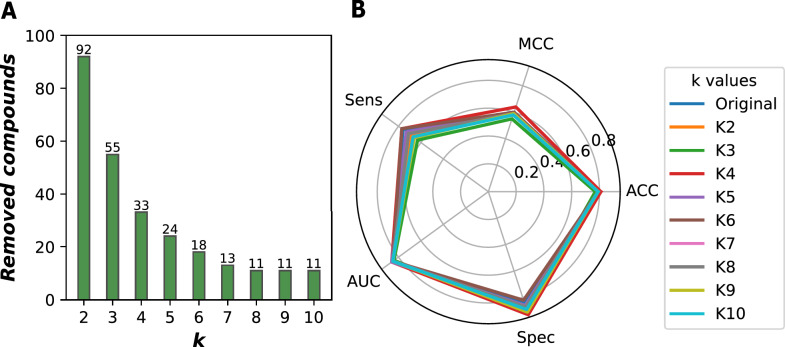
Performance evaluation of the LSTM model using within applicability domain compounds from the test set.** A **Number of removed compounds.** B **Evaluation metrics with various *k*-values

The model’s AD was thoroughly examined by systematically increasing *k* parameter from two to ten. The Euclidean distance-based *k* nearest neighbors techniques calculated the distance between each prediction and its *k* nearest training data points to refine AD assessment. This process effectively segregated out-of-domain chemicals from the independent test set, retaining only in-domain compounds for further analysis. Subsequently, our LSTM model was employed to predict outcomes for this optimized data set, allowing us to compare the refined performance metrics against the original values. The *k* value that yielded the most favorable evaluation metrics was identified as optimal. Fig7A illustrates the results of the AD assessment concerning skin irritation across various *k*-values.

The results derived from the model utilizing various *k* values are presented in Fig7B. The model employing a *k* value of four demonstrated the most robust predictive performance, attaining an accuracy of 0.814, a MCC of 0.639, a sensitivity of 0.768, an AUC of 0.858, and a specificity of 0.931. These performance metrics significantly outperformed those derived from other *k* values. At this optimal *k* setting, 33 out-of-domain chemicals-accounting for 18% of the test set-were excluded, leading to notable modifications in the performance metrics: an 1.6%, 6.1%, 1%, 1%, 11.2% improvement in accuracy, MCC, sensitivity, AUC, and specificity, respectively. The enhanced sensitivity and specificity underscore the model’s proficiency in accurately distinguishing between irritants and non-irritants. Furthermore, all adjusted metrics derived from the fine-tuned test set remained within the acceptable thresholds, thereby reinforcing the robustness of the model’s predictive performance.

### Key molecular structure related to skin irritation

The model-agnostic nature of permutation importance enables its application across diverse predictive models without the need for insight into or alteration of their internal components [50]. Consequently, it functioned as an effective tool for assessing the significance of features in our predictive model, specifically a LSTM network, utilizing conjoint features derived from MACCS keys and physicochemical descriptors.

**Fig. 8:**
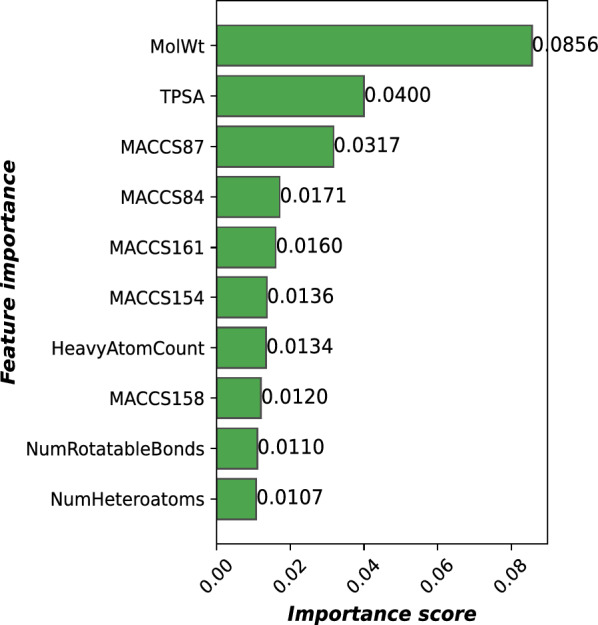
Feature importance for skin irritation prediction

In our analysis, we identified five physicochemical descriptors and five MACCS keys fingerprints as the top ten most importance features (Fig8). The key physicochemical descriptors included molecular weight (MolWt), topological polar surface area (TPSA), heavy atom count, number of rotatable bonds, and number of heteroatoms, ranking as the first, second, seventh, ninth, and tenth most important molecular features, respectively. For the MACCS fingerprints, the critical bits were 87, 84, 161, 154, and 158, corresponding to the third, fourth, fifth, sixth, and eighth most important features, respectively. Notably, the molecular weight emerged as the most influential, exerting the greatest impact on model performance and highlighting its significant role in predictive accuracy against skin irritation ability. Nevertheless, these results demonstrates that the model successfully utilized the structural insights from MACCS keys along with the broader molecular characteristics to improve its predictive capabilities. This interplay highlights the complex relationships presented in the data, demonstrating how various feature types worked together to enhance the model’s performance to predict outcomes accurately. Interpretations of the most important features are shown in the Table  1.

**Table 1:** Feature importance for skin irritation prediction

Feature importance	Description	SMARTS pattern
MolWt	Molecular weight	–
TPSA	Topological polar surface area	–
MACCS87	X!A$A	[F,Cl,Br,I]!@*@*
MACCS84	NH2	[NH2]
MACCS161	N	[#7]
MACCS154	C=O	[#6]=[#8]
HeavyAtomCount	Number of non-hydrogen atoms	–
MACCS158	C-N	[#6]-[#7]
NumRotatableBonds	Number of rotatable bonds	–
NumHeteroatoms	Number of heteroatoms	–

Molecular weight is widely regarded as a basic descriptor in predictive modeling, as it directly reflects molecular size, influencing essential properties like skin permeability [51]. Compounds with lower molecular weights are more likely to penetrate the skin’s protective barrier and reach deeper layers, intensifying their potential as chemical hazards [52]. Once absorbed, these substances can elicit a range of adverse effects, including skin irritation, sensitization, and systemic toxicity [53]. Low molecular weight organic chemicals can disrupt the integrity of the plasma membrane lipids, leading to the defatting and disintegration of the skin. This disruption results in skin irritation through the alteration of the skin’s barrier function and subsequent inflammatory responses [54]. Furthermore, the significant influence of molecular weight suggested that the model was identifying broad patterns related to molecular size instead of exploring complex chemical interactions. This observation indicated that more straightforward models or descriptors might provide similar insights, depending on the specific objectives of the study.

**Fig. 9:**
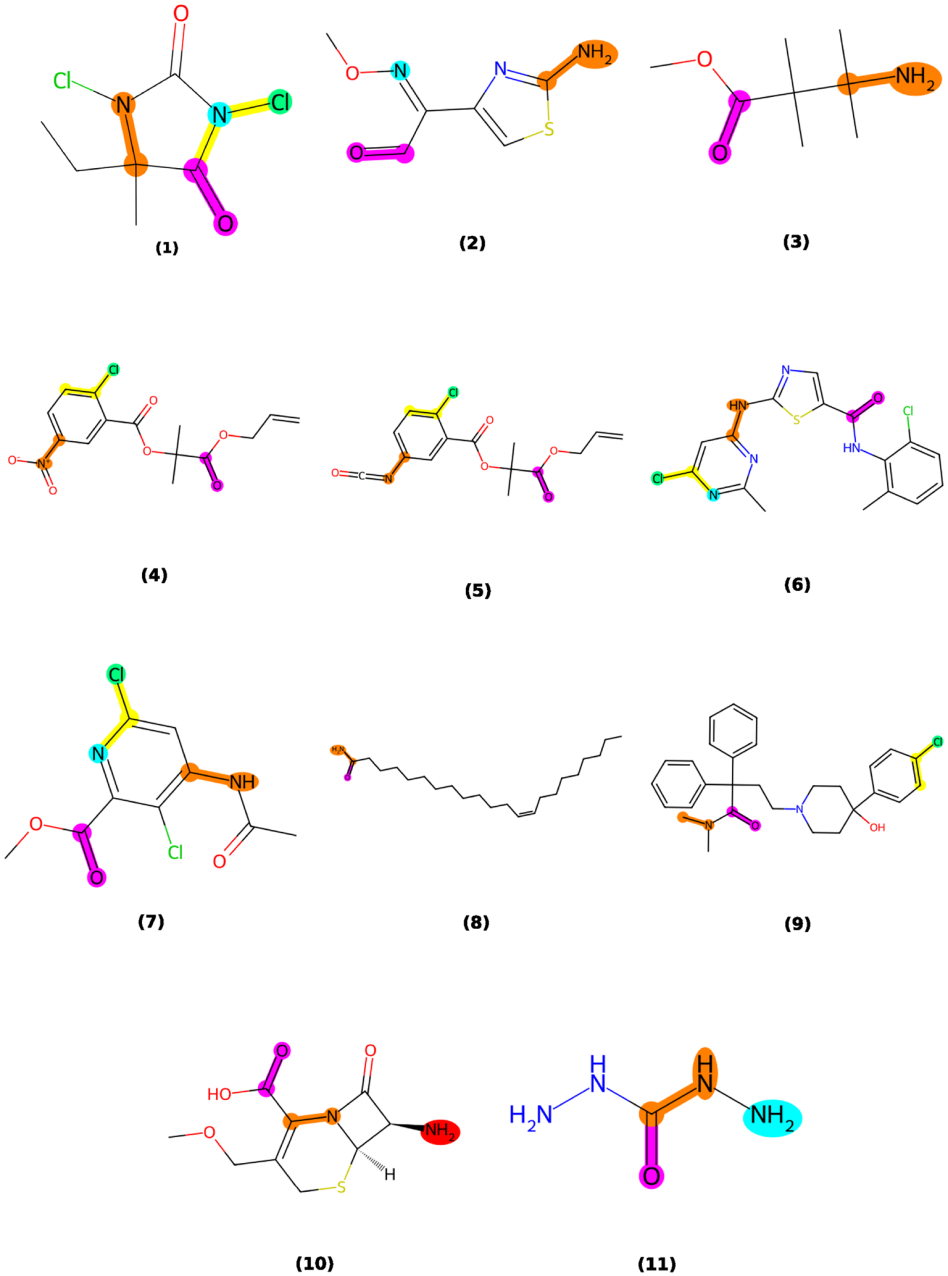
Compounds within the domain containing key substructures. Compounds (1-3) are identified as irritants, whereas compounds (4-11) are identified as non-irritants

Among the substructural features represented by the MACCS fingerprints, MACCS87 demonstrated the most significant influence, which is associated with the presence of halogen-containing substituents in the chemical structure [55]. Moreover, MACCS84, MACCS161, and MACCS158, corresponding to amine, nitrogen atom, and carbon attached to nitrogen atom, are also importance and correlated to the number of heavy atom and heteroatoms in the top ten importance features. Additionally, the MACCS154, which corresponding to the carbonyl group, is also importance for skin irritation classification. These results indicate that the presence of substituents containing halogen (MACCS87), oxygen (MACCS154), and nitrogen (MACCS84, MACCS161, and MACCS158) significantly impacts the performance of the predictive model. Interestingly, the electrophilic functional groups, which typically include atoms such as nitrogens, oxygens, or halogens bonded to a carbon atom, can generate a partial positive charge on the adjacent carbon atom. This alteration enhances the carbon atom’s reactivity toward electron-rich sites within peptides and proteins. Such increased reactivity may result in skin irritation by facilitating covalent interactions with skin proteins and disrupting the lipid components of the *stratum corneum* [54]. Particularly, Fig9 illustrates the example within-domain test compounds that contain significant substructures influencing the LSTM model for skin irritation prediction. Compounds (1-3) are identified as irritants, whereas compounds (4-11) are identified as non-irritants.

### Generalization performance of LSTM model based on other data sets

We further examine the model’s generalizability using another external compounds that not included in the training and the test sets. In this experiment, we sourced another external test molecules from several reputable databases, including the Australian Hazardous Chemical Information System, the European Registered Substances Factsheets, the New Zealand Chemical Classification and Information Database, the EU CLP Harmonized Classification, and ChemSkin. We ensured that these test molecules are not present in either the training or the test sets. Subsequently, we employed our LSTM model, utilizing conjoint features to predict the skin irritation risk associated with these compounds. The details of the compounds, including their names, CAS numbers, and prediction outcomes, are presented in Table  2. Furthermore, the predictive performance metrics obtained from this experiment are compiled in Table  3.

**Table 2:** Prediction outcomes for compounds beyond the training set of the LSTM model

CAS numbers	Predicting proability	Predicted label	Actual label
1000-78-8	0.9979	Irritant	Irritant
100-11-8	0.6330	Irritant	Irritant
100181-71-3	0.6038	Irritant	Irritant
10031-82-0	0.0044	Non-irritant	Irritant
100-38-9	0.9899	Irritant	Irritant
100-39-0	0.9401	Irritant	Irritant
100-40-3	0.0506	Non-irritant	Irritant
1072957-71-1	0.0007	Non-irritant	Non-irritant
1119-62-6	0.0191	Non-irritant	Non-irritant
1184-10-7	0.0037	Non-irritant	Non-irritant
118-82-1	0.0040	Non-irritant	Non-irritant
1263184-87-7	0.0055	Non-irritant	Non-irritant
139481-44-0	0.0008	Non-irritant	Non-irritant
201419-80-9	0.0318	Non-irritant	Non-irritant

The model demonstrated impressive predictive capabilities, achieving an accuracy of 85.7%, successfully classifying 12 out of 14 compounds. The measurement of sensitivity stood at 71.4%, reflecting the model’s ability to accurately identify true positive cases, successfully recognizing 5 out of 7 irritation compounds. The specificity achieved was 100%, showcasing the model’s effectiveness in identifying true negatives without any false positives, as illustrated by the correct classification of all 7 non-irritation compounds. Furthermore, the MCC and AUC values, recorded at 74.5% and 93.9%, respectively, provided additional evidence of the model’s remarkable discrimination capability.

**Table 3:** Predictive performance for external compounds beyond the LSTM model’s training and test sets

Accuracy (%)	MCC (%)	Sensitivity (%)	AUC (%)	Specificity (%)
85.7	74.5	71.4	93.9	100

### Performance comparison with existing models using their test sets

We further tested our model’s performance using external test sets from other methods. This experiment can demonstrate how well our model generalizes to different data sets. Moreover, it can elucidate our model’s performance compared to existing methods. In this experiment, we obtained test data sets from previous appropriate research and then applied the LSTM model to predict skin irritation. The data sets from STopTox, XGBoost, and the AttentiveSkin models were screened to exclude compounds present in the LSTM model training set prior to conducting the comparison. Table  4 explicates the effectiveness of the LSTM model compared to other predictive models [24, 56, 57].

**Table 4:** Performance comparison of LSTM model with other methods using their test sets

Models	Accuracy (%)	CCR (%)	MCC (%)	Sensitivity (%)	AUC (%)	Specificity (%)	F1 score (%)
Our LSTM using the StopTox	85.2	79.2	35.0	72.5	87.2	85.9	34.7
The StopTox	–	72.0	–	77.0	-	66.0	72.8
Our LSTM using the XGBoost test set	70.4	74.8	46.4	62.5	77.7	87.0	74.1
The XGBoost	73.4	66.9	–	82.1	-	51.6	81.5
Our LSTM using the AttentiveSkin test set	68.8	74.3	49.9	51.6	79.8	96.9	67.2
The AttentiveSkin	69.1	67.6	34.7	73.6	-	61.5	75.0

We found that the LSTM model exhibited superior performance compared to the STopTox model [56], achieving an accuracy of 85.2% and correct classification rate (CCR) of 79.2%, significantly outperforming STopTox model, which recorded a CCR of only 72.0%. In this context, CCR is defined as the arithmetic mean of sensitivity and specificity. The AUC metric also favored the LSTM model, which attained an impressive score of 87.2%, compared to the lack of AUC data from the STopTox model. The AUC represents the probability that a randomly chosen positive instance is ranked higher than a randomly chosen negative instance by the model. Although the LSTM model demonstrated marginally lower sensitivity compared to StopTox (72.5% vs. 77.0%, respectively), it exhibited substantially higher specificity, achieving 85.9% in contrast to the 66.0% specificity of the StopTox model. This indicates the superior ability of the LSTM model to accurately identify true negative cases, highlighting its robustness in minimizing false positive predictions. Although the STopTox model yielded a higher F1 score of 72.8%, its overall metrics highlighted limitations in predictive power. The reported metrics were obtained following the removal of duplicate compounds from the LSTM model training data set. The refined training set comprises 1,449 unique entries, consisting of 619 classified as irritants and 830 as non-irritants (Table  5). Collectively, these findings underscored the LSTM model’s robustness and reliability in predicting skin irritation, offering valuable insights for further research in this critical area of toxicology. It is important to note that StopTox serves as an alternative to traditional *in vivo* 6-pack tests, encompassing three topical and three systemic endpoints for assessing the toxicity hazard of small organic molecules. The LSTM outperforms the skin irritation prediction within StopTox but there is no evidence that it surpasses all other endpoints in this framework.

**Table 5:** The modified training data sets used to generate the comparative analysis

No	Models	Number of compounds	Irritants	Non-irritants
1	StopTox	1449	619	830
2	XGBoost	1651	560	1091
3	AttentiveSkin	1741	638	1103

When comparing the performance of our LSTM model with the XGBoost model [57], the LSTM model achieved a superior specificity of 87.0%, underscoring its capability to accurately classify non-irritant compounds-a critical characteristic for toxicity screening applications, as it helps to prevent the misclassification of non-harmful substances as irritants. In contrast, the XBGBoost model only had a specificity of 51.6% (Table  4). Additionally, the CCR of the LSTM model is higher than that from XGBoosting, 74.8% and 66.9%, respectively. However, the XGBoost model attained an higher accuracy at 73.4% and sensitivity at 82.1%, indicating its effectiveness in minimizing false negatives. The LSTM recorded the MCC of 46.4%, while the XGBoost model did not report this metric, which limited direct comparisons of their overall predictive performance. Furthermore, the LSTM model demonstrated a acceptable AUC of 77.7%, reflecting its effectiveness in ranking between irritant and non-irritant compounds, whereas the AUC for the XGBoost model was not available. An F1 score of 74.1% from the LSTM indicated the model is fairly good at predicting both positive and negative classes. However, its performance was lower than that of XGBoost, which attained an F1 score of 81.5%. These findings underscore the potential of the LSTM model to deliver reliable predictions regarding skin irritation, particularly in effectively identifying non-irritating compounds. In this analysis, the modified training set consists of 1,651 records, comprising 560 irritant samples and 1,091 non-irritant samples (Table  5).

Subsequently, we conducted a comparison of the performance between two models designed for skin irritation: the LSTM and the AttentiveSkin model [24] (Table  4). The revised training set for the LSTM model is made up 1,741 entries, with 638 designated as irritants and 1,103 categorized as non-irritants (Table  5). The LSTM model exhibits a marginally lower overall accuracy compared to the AttentiveSkin model, achieving 68.8% and 69.1%, respectively. However, in terms of CCR or balanced accuracy, the LSTM model demonstrates a superior performance, attaining 74.3%, whereas the AttentiveSkin model achieves 67.6%. Balanced accuracy is a performance metric that quantifies classification effectiveness by computing the mean of sensitivity (true positive rate) and specificity (true negative rate), thereby ensuring equitable evaluation across both classes. Unlike conventional accuracy, balanced accuracy remains unaffected by class distribution within the test set, making it particularly suitable for assessing QSAR models in cases of class imbalance [58]. Furthermore, the LSTM demonstrated a superior MCC of 49.9%, indicating a more robust correlation between predicted and actual values, whereas the AttentiveSkin model reached an MCC of merely 34.7%. The LSTM model exhibited a sensitivity of 51.6%, which was lower than the 73.6% achieved by the AttentiveSkin model. However, in terms of specificity, the LSTM outperformed AttentiveSkin, reaching 96.9% compared to 61.5%. This higher specificity highlights the LSTM model’s effectiveness in correctly identifying non-toxic compounds, thereby reducing false positive rates. Such a characteristic is crucial in screening applications, as it minimizes the misclassification of safe compounds as toxic, ensuring greater reliability in toxicity assessment. Additionally, the LSTM model achieved a notable AUC of approximately 80%, while the AUC for the AttentiveSkin model has not been reported. These results underscore the effectiveness of the LSTM model as a predictive instrument in toxicological evaluations, leading to improved assessments of chemical safety and refining the screening process for skin irritation risk in chemical substances.

## Discussion

Contact dermatitis is one of the most prevalent occupational illnesses, representing approximately 90-95% of all occupational skin disorders in the United States [59]. Acute dermatitis is characterized by symptoms such as itching, pain, redness, swelling, and the formation of a rash, with the potential for chronic changes, including altered pigmentation, skin thickening, and cracking due to repeated or prolonged exposure. Among its various forms, skin irritation, or irritant contact dermatitis (ICD), is the most common type of occupational skin disease, accounting for 70-80% of occupational contact dermatitis cases. ICD results from exposure to external hazardous agents that damage the skin’s barrier through non-immunological mechanisms. It can be triggered by acute exposure to highly irritating substances such as acids, bases, and oxidizing agents, or by cumulative chronic exposure to milder irritants like detergents and weak cleaning agents [60].

In the United States, the regulation of occupational skin exposure is governed by a comprehensive framework of at least 14 federal regulations, enforced by key agencies such as the Environmental Protection Agency (EPA), the U.S. FDA, and the Occupational Safety and Health Administration (OSHA) [61]. Notably, the National Institute for Occupational Safety and Health (NIOSH) issued “Current Intelligence Bulletin (CIB) 61: A Strategy for Assigning New NIOSH Skin Notations” in 2009, which provides an updated strategy for assigning skin notations [62]. The NIOSH Skin Notation (SK) profile provides information about the dermal absorption, corrosive, irritation, sensitization, and systemic toxicity of chemicals, are essential for determining the potential health hazards of substances resulting from skin exposure [63].

Additionally, cutaneous adverse drug reactions (CADRs) are a significant concern in drug research and development, as they encompass harmful skin effects triggered by drug use. These reactions can impact not only the skin but also its appendages, including nails, hair, and glands, highlighting the broad spectrum of potential adverse effects on the integumentary system. CADRs occur in 1-3% of adults and 2.5% of children treated with medications, impacting up to 10% of hospitalized patients. These skin reactions can be induced by any drug or regardless of administration route, whether over-the-counter, natural products, home remedies, or transdermal medications [64] [65]. For instance, a review of seven Transdermal Therapeutic Systems showed that 20%-50% of users experienced skin irritation [66]. Given the substantial health risks associated with skin irritation, it is essential to develop predictive models for assessing the toxicity of chemical compounds. Such models are critical for enabling accurate prediction, safeguarding human health and safety, and advancing drug development.

For that reason, we created and carefully evaluated a LSTM model for the computational prediction of skin irritation within a QSAR framework. This approach demonstrated exceptional predictive performance across multiple evaluation metrics, complemented by an in-depth examination of the model’s applicability domain. Furthermore, we conducted an comprehensive analysis of the molecular features that significantly influenced the model’s predictions, providing profound insights into the chemical properties that governed the potential for skin irritation.

The LSTM model that we developed, augmented with conjoint features including MACCS keys and physicochemical descriptors, exhibited strong predictive performance across a variety of data sets. By tackling the vanishing gradient issue found in conventional RNN architectures, LSTM stands out as an effective framework for handling sequential data, especially SMILES strings of chemical compounds. This adaptability highlights its potential for extensive practical applications in areas like natural product exploration, agricultural chemicals, and pharmaceutical development.

Feature importance quantifies the relative contribution of each predictor variable to the target outcome, serving as a fundamental tool for identifying data set characteristics and optimizing model performance. Feature importance analysis typically involves two primary methodologies: permutation importance [67] and SHAP (SHapley Additive Explanations) importance [68]. In this study, we adopted permutation feature importance due to its simplicity, model-agnostic nature, and ease of implementation. This method assesses feature relevance by measuring the reduction in model performance when a feature’s values are randomly shuffled, thereby breaking its association with the target variable. This approach aligns with our study’s objectives by providing an interpretable and transparent assessment of feature contributions. Alternatively, SHAP analysis offers a more nuanced interpretation by considering feature interactions and providing theoretically grounded attributions based on cooperative game theory [68]. However, SHAP is computationally expensive, particularly when applied to complex models such as our LSTM model, and may pose scalability challenges for large data sets. Given our focus on overall feature importance rather than instance-level explanations, permutation importance was the more practical choice. Despite this, we acknowledge the potential advantages of SHAP in offering deeper interpretability and plan to integrate SHAP-based analyses in future research to further enhance our understanding of feature importance.

## Limitations and future directions

This study contain some limitations that should be addressed in the further study. The primary limitation of our LSTM model is the extended training times and the restricted utilization of diverse molecular fingerprints, which hinder its capacity to thoroughly assess all molecular attributes related to chemical compounds. Specifically, the molecular features that we used in this study are limited to five individual sets of features, which may not capture all similarities between chemical structure; future studies may include a wider variety of molecular features to represent other aspect of molecular similarity for QSAR modeling. Secondly, we did not use a feature scaling function to preprocess the conjoint fingerprints, which may result in imbalanced gradient optimization and lead to longer training times. In this study, explicit feature scaling or normalization was not applied to balance these descriptors. Instead, we relied on the inherent adaptability of DL algorithms to automatically learn appropriate weights for each feature type during training. The RNN models, such as the GRU and LSTM architectures employed in this work, are armed to address differences in feature ranges through mechanisms like weight optimization during back-propagation, enabling the model to assign appropriate importance to both continuous and binary features without explicit scaling [69]. However, future work should explore feature scaling or weighting strategies to address potential imbalances better and improve the robustness of applicability domain assessments.

Nevertheless, this approach still demonstrated significant potential in predicting skin irritation, indicating several promising avenues for future research. A primary opportunity involves expanding the model’s applicability domain by integrating diverse data sets, thereby enhancing generalizability and predictive performance and providing a more nuanced understanding of the mechanisms underlying skin irritation across various compounds. Furthermore, exploring hybrid models that combine the strengths of LSTM with advanced machine learning techniques, such as ensemble methods, holds considerable promise for refining predictions and improving both accuracy and interpretability. By leveraging the unique advantages of multiple methodologies, future research could establish a more robust and comprehensive framework for toxicity prediction, ultimately facilitating the development of safer products. Nevertheless, future research should employ SHAP analysis to attain a more comprehensive understanding of feature importance.

## Conclusion

In conclusion, our LSTM model demonstrated strong predictive capabilities for skin irritation, characterized by high accuracy and a well-defined applicability domain. The identification of important features not only elucidates potential mechanisms of action but also provides a foundation for further skin irritation investigations. Given the increasing societal and regulatory emphasis on reducing reliance on animal testing, our model represents a significant advancement in the field of predictive toxicology. It provides a powerful alternative for early-stage screening of chemical compounds, significantly contributing to safer drug development and chemical manufacturing.

## Additional file

Supplementary file 1.

Supplementary file 2.

## Data Availability

All data used in this study are available in the Supporting Information.

## Abbreviations

AD: Applicability domain
BiGRU: Bidirectional gated recurrent unit
BiLSTM: Bidirectional long short-term memory
CADRs: Cutaneous adverse drug reactions
EPA: U.S. environmental protection agency
GRU: Gated recurrent unit
ICCVAM: Interagency coordinating committee on the validation of alternative methods
ICD: Irritant contact dermatitis
LSTM: Long short-term memory
NIOSH: National institute for occupational safety and health
OECD: Organisation for economic co-operation and development
QSAR: Quantitative structure-activity relationship
RNN: Recurrent neural network
ROC-AUC: Area under the receiver operating characteristic
SMILES: Simplified molecular-input line entry system
MCC: Matthews correlation coefficient

## Code availability

The script to reproduce this analysis can be downloaded from the following URL: https://github.com/taraponglab/irritationrnn.
