# Supplementary material for: Protecting your skin: a highly accurate LSTM network integrating conjoint features for predicting chemical-induced skin irritation
Source: J Cheminform. 2025 Mar 27;17:39. doi: 10.1186/s13321-025-00980-y (PMC11951793; doi:10.1186/s13321-025-00980-y)
Supplement: Supplementary file 1 [file 13321_2025_980_MOESM1_ESM.pdf]

Supplementary Information

Protecting your skin: a highly accurate LSTM network integrating conjoint features for predicting chemical-induced skin irritation

Huynh Anh Duy, Tarapong Srisongkram*

[1] Graduate School in the Program in Research and Development in Pharmaceuticals, Faculty of Pharmaceutical Sciences, Khon Kaen University, Khon Kaen, 40002, Thailand.

[1] Division of Pharmaceutical Chemistry, Faculty of Pharmaceutical Sciences, Khon Kaen University, Khon Kaen, 40002, Thailand

*** Corresponding author**

Email: tarasri@kku.ac.th

Table of content

Supplementary Table 1. The full evaluation model performance of all models……2

Supplementary Table 1. The full evaluation model performance of all models

No.	RNN-based models for skin irritation prediction	Accuracy	MCC	Sensitivity	AUC	Specificity
1	simpleRNN_Tokens	0.624	0.242	0.459	0.694	0.771
2	simpleRNN_Tokens	0.619	0.23	0.482	0.693	0.74
3	simpleRNN_Tokens	0.635	0.268	0.447	0.694	0.802
4	simpleRNN_RDKit_Phychem	0.718	0.443	0.553	0.779	0.865
5	simpleRNN_RDKit_Phychem	0.729	0.464	0.576	0.809	0.865
6	simpleRNN_RDKit_Phychem	0.702	0.408	0.529	0.798	0.854
7	simpleRNN_RDKit	0.702	0.406	0.541	0.778	0.844
8	simpleRNN_RDKit	0.702	0.406	0.541	0.756	0.844
9	simpleRNN_RDKit	0.696	0.398	0.518	0.788	0.854
10	simpleRNN_Phychem	0.713	0.425	0.588	0.822	0.823
11	simpleRNN_Phychem	0.713	0.463	0.447	0.819	0.948
12	simpleRNN_Phychem	0.707	0.434	0.482	0.805	0.906
13	simpleRNN_MACCS_RDKit	0.718	0.443	0.553	0.795	0.865
14	simpleRNN_MACCS_RDKit	0.735	0.474	0.588	0.801	0.865
15	simpleRNN_MACCS_RDKit	0.707	0.417	0.553	0.792	0.844
16	simpleRNN_MACCS_Phychem	0.757	0.526	0.588	0.841	0.906
17	simpleRNN_MACCS_Phychem	0.773	0.548	0.671	0.844	0.865
18	simpleRNN_MACCS_Phychem	0.796	0.604	0.647	0.877	0.927
19	simpleRNN_MACCS	0.751	0.501	0.671	0.828	0.823
20	simpleRNN_MACCS	0.779	0.558	0.694	0.836	0.854
21	simpleRNN_MACCS	0.779	0.558	0.694	0.836	0.854
22	simpleRNN_ECFP_RDKit	0.729	0.472	0.541	0.813	0.896
23	simpleRNN_ECFP_RDKit	0.718	0.448	0.529	0.808	0.885
24	simpleRNN_ECFP_RDKit	0.718	0.445	0.541	0.817	0.875
25	simpleRNN_ECFP_Phychem	0.685	0.376	0.494	0.771	0.854
26	simpleRNN_ECFP_Phychem	0.702	0.408	0.529	0.77	0.854
27	simpleRNN_ECFP_Phychem	0.674	0.354	0.471	0.777	0.854
28	simpleRNN_ECFP_MACCS	0.674	0.374	0.4	0.788	0.917
29	simpleRNN_ECFP_MACCS	0.674	0.366	0.424	0.786	0.896
30	simpleRNN_ECFP_MACCS	0.685	0.401	0.412	0.8	0.927
31	simpleRNN_ECFP	0.685	0.376	0.494	0.768	0.854
32	simpleRNN_ECFP	0.696	0.4	0.506	0.764	0.865
33	simpleRNN_ECFP	0.685	0.374	0.506	0.766	0.844
34	LSTM_Tokens	0.624	0.242	0.482	0.672	0.75
35	LSTM_Tokens	0.619	0.236	0.388	0.69	0.823
36	LSTM_Tokens	0.657	0.31	0.529	0.699	0.771
37	LSTM_RDKit_Phychem	0.751	0.513	0.588	0.829	0.896
38	LSTM_RDKit_Phychem	0.746	0.5	0.588	0.827	0.885
39	LSTM_RDKit_Phychem	0.762	0.534	0.612	0.824	0.896
40	LSTM_RDKit	0.674	0.366	0.424	0.758	0.896
41	LSTM_RDKit	0.718	0.443	0.553	0.793	0.865
42	LSTM_RDKit	0.707	0.417	0.553	0.804	0.844
43	LSTM_Phychem	0.685	0.371	0.529	0.773	0.823

No.	RNN-based models for skin irritation prediction	Accuracy	MCC	Sensitivity	AUC	Specificity
44	LSTM_Phychem	0.729	0.459	0.612	0.798	0.833
45	LSTM_Phychem	0.646	0.295	0.435	0.745	0.833
46	LSTM_MACCS_RDKit	0.746	0.506	0.565	0.814	0.906
47	LSTM_MACCS_RDKit	0.707	0.442	0.459	0.779	0.927
48	LSTM_MACCS_RDKit	0.757	0.521	0.612	0.808	0.885
49	LSTM_MACCS_Phychem	0.785	0.567	0.753	0.845	0.812
50	LSTM_MACCS_Phychem	0.851	0.705	0.765	0.867	0.927
51	LSTM_MACCS_Phychem	0.768	0.535	0.765	0.836	0.771
52	LSTM_MACCS	0.768	0.538	0.659	0.841	0.865
53	LSTM_MACCS	0.773	0.552	0.647	0.848	0.885
54	LSTM_MACCS	0.807	0.617	0.706	0.834	0.896
55	LSTM_ECFP_RDKit	0.702	0.432	0.447	0.687	0.927
56	LSTM_ECFP_RDKit	0.652	0.322	0.376	0.636	0.896
57	LSTM_ECFP_RDKit	0.674	0.374	0.4	0.658	0.917
58	LSTM_ECFP_Phychem	0.680	0.368	0.471	0.8	0.865
59	LSTM_ECFP_Phychem	0.657	0.333	0.388	0.799	0.896
60	LSTM_ECFP_Phychem	0.691	0.389	0.494	0.801	0.865
61	LSTM_ECFP_MACCS	0.674	0.357	0.459	0.662	0.865
62	LSTM_ECFP_MACCS	0.685	0.381	0.471	0.673	0.875
63	LSTM_ECFP_MACCS	0.68	0.37	0.459	0.667	0.875
64	LSTM_ECFP	0.68	0.365	0.482	0.771	0.854
65	LSTM_ECFP	0.674	0.366	0.424	0.769	0.896
66	LSTM_ECFP	0.652	0.312	0.412	0.765	0.865
67	GRU_Tokens	0.591	0.174	0.353	0.682	0.802
68	GRU_Tokens	0.619	0.233	0.412	0.687	0.802
69	GRU_Tokens	0.624	0.245	0.424	0.673	0.802
70	GRU_RDKit_Phychem	0.746	0.514	0.541	0.834	0.927
71	GRU_RDKit_Phychem	0.757	0.526	0.588	0.816	0.906
72	GRU_RDKit_Phychem	0.757	0.524	0.6	0.833	0.896
73	GRU_RDKit	0.718	0.443	0.553	0.782	0.865
74	GRU_RDKit	0.691	0.389	0.494	0.743	0.865
75	GRU_RDKit	0.696	0.403	0.494	0.795	0.875
76	GRU_Phychem	0.762	0.524	0.671	0.81	0.844
77	GRU_Phychem	0.724	0.458	0.541	0.807	0.885
78	GRU_Phychem	0.685	0.367	0.565	0.752	0.792
79	GRU_MACCS_RDKit	0.724	0.456	0.553	0.795	0.875
80	GRU_MACCS_RDKit	0.735	0.477	0.576	0.798	0.875
81	GRU_MACCS_RDKit	0.718	0.445	0.541	0.793	0.875
82	GRU_MACCS_Phychem	0.74	0.48	0.635	0.831	0.833
83	GRU_MACCS_Phychem	0.796	0.616	0.612	0.838	0.958
84	GRU_MACCS_Phychem	0.834	0.669	0.765	0.874	0.896
85	GRU_MACCS	0.807	0.619	0.694	0.851	0.906
86	GRU_MACCS	0.801	0.602	0.729	0.847	0.865
87	GRU_MACCS	0.757	0.512	0.682	0.808	0.823

No.	RNN-based models for skin irritation prediction	Accuracy	MCC	Sensitivity	AUC	Specificity
88	GRU_ECFP_RDKit	0.696	0.422	0.435	0.792	0.927
89	GRU_ECFP_RDKit	0.751	0.528	0.541	0.813	0.938
90	GRU_ECFP_RDKit	0.735	0.493	0.518	0.826	0.927
91	GRU_ECFP_Phychem	0.674	0.366	0.424	0.811	0.896
92	GRU_ECFP_Phychem	0.663	0.33	0.459	0.801	0.844
93	GRU_ECFP_Phychem	0.68	0.368	0.471	0.801	0.865
94	GRU_ECFP_MACCS	0.68	0.355	0.588	0.714	0.76
95	GRU_ECFP_MACCS	0.591	0.174	0.494	0.653	0.677
96	GRU_ECFP_MACCS	0.68	0.377	0.435	0.731	0.896
97	GRU_ECFP	0.68	0.361	0.506	0.78	0.833
98	GRU_ECFP	0.652	0.322	0.376	0.779	0.896
99	GRU_ECFP	0.663	0.332	0.447	0.756	0.854
100	BiLSTM_Tokens	0.602	0.197	0.4	0.651	0.781
101	BiLSTM_Tokens	0.602	0.197	0.4	0.669	0.781
102	BiLSTM_Tokens	0.597	0.184	0.412	0.64	0.76
103	BiLSTM_RDKit_Phychem	0.729	0.466	0.565	0.796	0.875
104	BiLSTM_RDKit_Phychem	0.746	0.51	0.553	0.815	0.917
105	BiLSTM_RDKit_Phychem	0.751	0.51	0.6	0.828	0.885
106	BiLSTM_RDKit	0.724	0.465	0.518	0.746	0.906
107	BiLSTM_RDKit	0.702	0.411	0.518	0.774	0.865
108	BiLSTM_RDKit	0.729	0.464	0.576	0.772	0.865
109	BiLSTM_Phychem	0.718	0.434	0.624	0.779	0.802
110	BiLSTM_Phychem	0.702	0.403	0.565	0.799	0.823
111	BiLSTM_Phychem	0.702	0.423	0.471	0.805	0.906
112	BiLSTM_MACCS_RDKit	0.729	0.479	0.518	0.779	0.917
113	BiLSTM_MACCS_RDKit	0.735	0.474	0.588	0.787	0.865
114	BiLSTM_MACCS_RDKit	0.707	0.434	0.482	0.771	0.906
115	BiLSTM_MACCS_Phychem	0.757	0.548	0.529	0.852	0.958
116	BiLSTM_MACCS_Phychem	0.807	0.615	0.718	0.855	0.885
117	BiLSTM_MACCS_Phychem	0.79	0.597	0.624	0.853	0.938
118	BiLSTM_MACCS	0.801	0.603	0.718	0.831	0.875
119	BiLSTM_MACCS	0.746	0.496	0.612	0.837	0.865
120	BiLSTM_MACCS	0.779	0.559	0.682	0.837	0.865
121	BiLSTM_ECFP_RDKit	0.707	0.438	0.471	0.789	0.917
122	BiLSTM_ECFP_RDKit	0.751	0.52	0.565	0.81	0.917
123	BiLSTM_ECFP_RDKit	0.735	0.498	0.506	0.797	0.938
124	BiLSTM_ECFP_Phychem	0.663	0.341	0.412	0.795	0.885
125	BiLSTM_ECFP_Phychem	0.646	0.343	0.294	0.785	0.958
126	BiLSTM_ECFP_Phychem	0.663	0.335	0.435	0.809	0.865
127	BiLSTM_ECFP_MACCS	0.702	0.406	0.541	0.783	0.844
128	BiLSTM_ECFP_MACCS	0.669	0.334	0.529	0.702	0.792
129	BiLSTM_ECFP_MACCS	0.635	0.265	0.553	0.698	0.708
130	BiLSTM_ECFP	0.663	0.33	0.459	0.771	0.844
131	BiLSTM_ECFP	0.635	0.278	0.376	0.775	0.865

No.	RNN-based models for skin irritation prediction	Accuracy	MCC	Sensitivity	AUC	Specificity
132	BiLSTM_ECFP	0.635	0.272	0.412	0.758	0.833
133	BiGRU_Tokens	0.619	0.236	0.388	0.657	0.823
134	BiGRU_Tokens	0.602	0.2	0.353	0.671	0.823
135	BiGRU_Tokens	0.619	0.236	0.388	0.648	0.823
136	BiGRU_RDKit_Phychem	0.762	0.544	0.576	0.821	0.927
137	BiGRU_RDKit_Phychem	0.757	0.526	0.588	0.83	0.906
138	BiGRU_RDKit_Phychem	0.746	0.503	0.576	0.82	0.896
139	BiGRU_RDKit	0.74	0.49	0.576	0.808	0.885
140	BiGRU_RDKit	0.696	0.403	0.494	0.79	0.875
141	BiGRU_RDKit	0.702	0.416	0.494	0.777	0.885
142	BiGRU_Phychem	0.696	0.389	0.6	0.76	0.781
143	BiGRU_Phychem	0.713	0.43	0.553	0.799	0.854
144	BiGRU_Phychem	0.68	0.358	0.529	0.757	0.812
145	BiGRU_MACCS_RDKit	0.713	0.441	0.506	0.778	0.896
146	BiGRU_MACCS_RDKit	0.713	0.444	0.494	0.762	0.906
147	BiGRU_MACCS_RDKit	0.702	0.416	0.494	0.777	0.885
148	BiGRU_MACCS_Phychem	0.801	0.621	0.635	0.859	0.948
149	BiGRU_MACCS_Phychem	0.773	0.582	0.553	0.858	0.969
150	BiGRU_MACCS_Phychem	0.779	0.567	0.635	0.856	0.906
151	BiGRU_MACCS	0.768	0.538	0.659	0.812	0.865
152	BiGRU_MACCS	0.751	0.508	0.612	0.807	0.875
153	BiGRU_MACCS	0.74	0.482	0.624	0.826	0.844
154	BiGRU_ECFP_RDKit	0.724	0.456	0.553	0.792	0.875
155	BiGRU_ECFP_RDKit	0.718	0.463	0.482	0.788	0.927
156	BiGRU_ECFP_RDKit	0.707	0.442	0.459	0.797	0.927
157	BiGRU_ECFP_Phychem	0.663	0.335	0.435	0.8	0.865
158	BiGRU_ECFP_Phychem	0.691	0.389	0.494	0.798	0.865
159	BiGRU_ECFP_Phychem	0.68	0.365	0.482	0.812	0.854
160	BiGRU_ECFP_MACCS	0.663	0.33	0.459	0.728	0.656
161	BiGRU_ECFP_MACCS	0.652	0.299	0.588	0.725	0.708
162	BiGRU_ECFP_MACCS	0.68	0.362	0.706	0.754	0.844
163	BiGRU_ECFP	0.652	0.312	0.412	0.772	0.865
164	BiGRU_ECFP	0.669	0.343	0.459	0.769	0.854
165	BiGRU_ECFP	0.646	0.297	0.424	0.773	0.844
